# Comparative Evaluation of Proprietary and Open-Source Large Language Models for Systematic Multi-source Information Extraction in Interventional Oncology

**DOI:** 10.1007/s00270-025-04287-1

**Published:** 2025-12-07

**Authors:** Elif Can, Wibke Uller, Elmar Kotter, Katharina Vogt, Michael Doppler, Michael Brönnimann, Raid Alshinibr, Aboelyazid Elkilany, Felix Busch, Avan Kader, Sebastian Gassenmaier, Saif Afat, Marcus R. Makowski, Keno K. Bressem, Lisa C. Adams

**Affiliations:** 1 Department of Diagnostic and Interventional Radiology, Medical Center – University of Freiburg, Faculty of Medicine, University of Freiburg, Hugstetter Str. 55, 79106 Freiburg, Germany; 2 Department of Diagnostic, Interventional and Pediatric Radiology, Inselspital, Bern University Hospital, University of Bern, Bern, Switzerland; 3 Department of Diagnostic and Interventional Radiology, Charité – Universitätsmedizin Berlin, corporate member of Freie Universität Berlin and Humboldt Universität zu Berlin, Berlin, Germany; 4 Department of Diagnostic and Interventional Radiology, University Hospital Leipzig, Leipzig, Saxony Germany; 5 Department of Radiology, Klinikum rechts der Isar, Technical University of Munich (TUM), Munich, Germany; 6 Department of Diagnostic and Interventional Radiology, University Hospital Tübingen, Tübingen, Germany

**Keywords:** Hepatocellular carcinoma, Transarterial chemoembolization, Interventional radiology, Large language models, Artificial intelligence, Natural language processing, Longitudinal clinical data, Structured reporting

## Abstract

**Purpose:**

To compare proprietary (GPT-4o, Gemini 1.5 Pro) and open-source (Llama 3.1 70B, Llama 3.1 405B) large language models (LLMs) for extracting clinically relevant variables from transarterial chemoembolization (TACE) reports in patients with hepatocellular carcinoma (HCC).

**Methods:**

Retrospective analysis of 556 anonymized longitudinal TACE-related reports (radiology, interventional procedure, and clinical follow-up) from 50 patients with HCC treated between 2012 and 2024 at a single tertiary center was carried out. Models extracted predefined binary variables (e.g., modified Response Evaluation Criteria in Solid Tumors [mRECIST] tumor response, alpha-fetoprotein [AFP] dynamics, Barcelona Clinic Liver Cancer [BCLC] stage) and ordinal variables (e.g., liver segment involvement, vascular invasion, follow-up assessment) using a standardized system prompt and output template. Model performance was assessed by accuracy, ordinal scores, and longitudinal error rates using mixed-effects regression with patient-level random intercepts.

**Results:**

Proprietary models outperformed open-source models. GPT-4o and Gemini achieved the highest mean accuracies for binary variables (0.87 ± 0.21 and 0.85 ± 0.16) and ordinal variables (4.15/5 and 4.10/5), significantly exceeding both Llama models (*p < *0.05). GPT-4o showed the lowest longitudinal error rate for binary variables (0.01 vs 0.09–0.21 for the other models), indicating greater robustness over time. All models showed poor performance in vascular invasion detection and follow-up assessment.

**Conclusion:**

Proprietary LLMs can accurately extract most key TACE-related variables from routine clinical reports and may support decision-making in interventional oncology; however, all models showed poor performance in vascular invasion detection and follow-up assessment, so expert human oversight remains essential.

**Graphical abstract:**

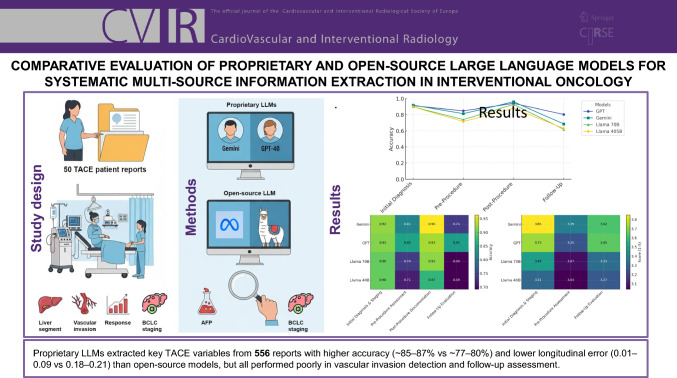

**Supplementary Information:**

The online version contains supplementary material available at 10.1007/s00270-025-04287-1.

## Introduction

Effective decision-making in interventional oncology is critical for managing complex conditions such as hepatocellular carcinoma (HCC), a highly aggressive and increasingly prevalent liver cancer often necessitating multidisciplinary care. With the rising incidence of HCC driven by chronic liver diseases, such as hepatitis and nonalcoholic fatty liver disease, precise and data-driven treatment planning has become indispensable. Transarterial chemoembolization (TACE) remains a cornerstone therapy for intermediate-stage HCC, offering a minimally invasive, dual-action approach that combines targeted chemotherapy with embolization to disrupt the tumor’s blood supply, and is associated with improved survival and quality-of-life outcomes (1–4). The success of TACE relies heavily on thorough pre-procedural planning, precise execution, and meticulous post-procedural monitoring (3–6). Key clinical assessments, including imaging for segmental liver involvement (7), detection of vascular invasion (8), alpha-fetoprotein (AFP) biomarker trends (9, 10), and evaluation of tumor response using the modified Response Evaluation Criteria in Solid Tumors (mRECIST)(11), are integral to determining treatment eligibility and guiding subsequent decisions. These decisions are typically guided by multidisciplinary tumor boards, underscoring the complexity and collaborative approach essential to the effective management of HCC (1, 4–6). Despite its clinical importance, interpreting TACE-related data remains challenging due to the volume, complexity, and multimodal nature of information, spanning imaging, procedural, and laboratory findings, as well as variability in clinician interpretation. These factors can introduce inconsistencies and delay critical decisions. Automated systems that reliably extract and synthesize relevant clinical information have the potential to alleviate these challenges. Recent advancements in artificial intelligence (AI), particularly large language models (LLMs), have demonstrated promising capabilities in processing unstructured medical texts. Proprietary LLMs, such as GPT-4o and Gemini 1.5 Pro, have shown particular success in extracting structured clinical information and streamlining workflows in radiology and oncology (12–23). However, their performance specifically within radiology and interventional oncology remains insufficiently understood (12–14).

This study aimed to comparatively evaluate proprietary (GPT-4o, Gemini 1.5 Pro) and open-source (Llama 3.1 70B, Llama 3.1 405B) LLMs regarding their effectiveness in automating the extraction of key clinical variables from TACE reports and accompanying longitudinal tabular data. Automating this extraction could significantly facilitate clinical decision-making, particularly in complex scenarios requiring clinicians to analyze multiple reports and longitudinal laboratory trends. This exploratory benchmarking analysis systematically investigates the strengths and limitations of current LLMs, providing insights into their potential for future clinical integration, but without asserting immediate readiness for implementation.

## Methods

### Study Design and Data Collection

This retrospective study analyzed 556 anonymized reports from 50 HCC patients treated consecutively between January 2012 and December 2024 at a German referral center. Each patient contributed an average of 11 text-based clinical reports, including radiology reports (e.g., computed tomography (CT) and magnetic resonance imaging (MRI) interpretation), interventional procedure reports, and follow-up clinical summaries authored by radiologists and interventional radiologists. Multidisciplinary tumor board notes were not included. Only original source documentation, not synthesized summary documents, was used to ensure unstructured, clinically realistic model input. The number of TACE sessions per patient varied depending on individual treatment needs.

Inclusion criteria for patient reports were clearly documented clinical variables critical to HCC management: mRECIST tumor response assessments, AFP levels, and vascular invasion status. Vascular invasion was strictly defined as tumor thrombosis within the portal or hepatic veins, explicitly excluding non-thrombotic vascular abnormalities such as arterioportal fistulas to ensure clinical precision. Reports lacking documentation of these variables or containing ambiguous findings were excluded to maintain data integrity and analytical consistency. Tabular data, including AFP trends and liver function parameters, were extracted from the hospital’s information system to supplement narrative report content. To account for variability in clinical documentation styles over the 13-year study period, standardized preprocessing was systematically applied prior to analysis by the large language models (LLMs). Preprocessing included unifying section headings (e.g., procedure details, imaging findings, follow-up notes), normalizing medical terminology (e.g., consistent abbreviations and terminology), removing institutional formatting artifacts, and standardizing temporal references (e.g., converting relative time statements such as “two weeks ago” into absolute calendar dates). These standardizations were implemented to create uniform semantic structures across reports, thereby enhancing model interpretability and performance. Reports lacking required clinical variables were excluded prior to analysis according to predefined criteria.

The study was approved by the local ethics committee (Nr. 25-1032-S1-retro), and all patient data were fully anonymized to ensure confidentiality and ethical compliance.

## Model Selection

Four LLMs were evaluated: two proprietary models, Gemini 1.5 Pro (Gemini) and GPT-4o (GPT), and two open-source models, Llama 3.1 70B and Llama 3.1 405B (24, 25). Llama 3.1 405B is open-source with weights available. While other open-source models such as Mistral and Phi could provide valuable comparisons, we selected Llama models based on their strong general language understanding capabilities and established performance in medical text extraction benchmarks (25, 26). Future studies should include these alternative models to determine whether their specific strengths translate to improved performance in clinical information extraction tasks (12, 27). Key variables for assessment included liver segment involvement, vascular invasion, mRECIST response, AFP trends, and Barcelona Clinic Liver Cancer (BCLC) staging, all crucial for decision-making in TACE workflows. The models’ performance in accurately identifying and interpreting these variables was central to evaluating their potential to enhance clinical efficiency and decision support.

## Data Extraction and Integration

Textual and laboratory data were fully anonymized before analysis. Each model independently processed narrative and tabular data, subsequently integrating these outputs into a comprehensive clinical patient profile. The prompt, including detailed instructions and the standardized TACE Report Template, was uniformly applied to all four models without individual adjustments (**Supplement 1**). All prompts were implemented as system-role instructions to ensure consistent context priming across models; no mixture of user and system roles was used. Temperature was set to 0 for all runs. All models adhered to the predefined output template; only minor lexical variations (e.g., synonyms, punctuation) occurred, without structural deviation. No additional prompt engineering or model optimization (e.g., adjustment of temperature, top-k, or top-p parameters) was performed, ensuring an unbiased assessment of the models’ baseline capabilities under realistic clinical conditions. A fixed random seed (42) was applied for GPT-4o and Llama models that support deterministic seeding.

Temporal synchronization between tabular and narrative data was achieved by aligning timestamps, enabling the models to contextualize narrative findings alongside quantitative laboratory trends. This standardized and minimally adjusted approach ensured consistency, reproducibility, and comparability across all evaluated models, reflecting practical deployment conditions in clinical workflows. AFP time series were aligned to report timestamps using the nearest-neighbor method within a ± 14-day window. Missing values were linearly interpolated when adjacent data points were available; ties were resolved by the earliest date. Model accuracies for AFP trend interpretation were compared using mixed-effects logistic regression with model as a fixed effect and patient as a random intercept.

## Evaluation of Clinical Variables

Ground truth for each clinical variable was established by an expert radiologist with over 9 years of experience (E.C.). To ensure scoring consistency and address potential bias from a single-rater design, a second board-certified radiologist (L.C.A, 9 years of experience) independently evaluated the entire dataset. The same predefined scoring rubric was applied. Both raters were blinded to each other’s annotations. Disagreements between raters were resolved through consensus discussion, with consultation of a third board-certified radiologist (9 years of experience) when consensus could not be reached. The consensus process was documented, including the reason for disagreement and resolution rationale. Liver segment involvement and vascular invasion were scored on a 1–5 scale, reflecting clinical relevance. For tasks requiring sequential data interpretation (e.g., AFP trends), accuracy was assessed both per instance and longitudinally. The rationale for selecting specific performance metrics, such as ordinal scoring for liver segment involvement, was based on the need to capture clinical nuance and variability in task complexity. Ordinal scoring provides a more detailed and clinically nuanced evaluation compared to binary metrics, which were used to calculate overall accuracy, allowing for differentiation between partial and complete successes in variable extraction. This approach ensures a detailed and clinically relevant evaluation of model performance.

## Task Categorization Across TACE Phases

The evaluation of AI model performance was structured across four key phases of TACE patient care, each with specific clinical tasks critical to patient management: (1) Initial Diagnosis and Staging: Diagnosis Accurate (1/0), Initial Diagnosis Date Accurate (1/0), Exam Dates Chronological (1/0), Liver Segments Accurately Identified (1–5), Vascular Involvement Correctly Reported (1–5); (2) Pre-Procedure Assessment: Lymph Node Status Accurately Described (1–5), Distant Metastases Identified (1–5), BCLC Stage Correct (1/0), Child–Pugh Score Correct (1/0), TACE Procedure Dates Accurate (1/0), TACE Type Consistency (1/0); (3) Post-Procedure Documentation: Target Lesions Correctly Identified (1/0), Lipiodol Retention Accurately Reported (1/0), TACE Complications Accurately Reported (1/0), mRECIST Assessment Correct (1/0); (4) Follow-Up Evaluation: AFP Trend Accurately Reported (1/0), Time to Progression Correctly Calculated (1/0), Accuracy of Findings Across Time Points (1–5), Key Changes Summary Correct (1–5), Liver Function Trends Accurately Reported (1–5), TACE Suitability Assessment Appropriate (1/0), Alternative Treatments Appropriately Suggested (1/0), Follow-Up Imaging Recommendations Appropriate (1/0).

Tasks in the Initial Diagnosis and Staging and Pre-Procedure Assessment phases emphasized structured binary outcomes (e.g., accuracy of staging or dates). In contrast, tasks in the Post-Procedure Documentation and Follow-Up Evaluation phases required both binary evaluations (e.g., mRECIST assessment) and interpretive, ordinal metrics (e.g., liver function trends, key changes summary). This categorization facilitated a systematic evaluation of the models’ strengths and weaknesses across both simple and complex tasks.

## Statistical Analysis

Descriptive statistics were used to summarize model accuracy across all clinical variables, including liver segment involvement, vascular invasion, mRECIST tumor response, and AFP trends. Performance metrics were calculated at the report level and aggregated by clinical variable and model.

Statistical analyses were conducted using R software (version 4.2.3), with supplementary data processing and visualization performed in Python (version 3.10, using libraries such as pandas and matplotlib). Group differences were examined with generalized linear mixed-effects models that included a random intercept for patient and a fixed effect for the model. Post hoc contrasts were evaluated with Tukey-adjusted Wald tests.

A longitudinal error analysis was performed to evaluate model consistency across temporally ordered reports. For each patient, intra-patient variability in model accuracy was quantified by calculating the standard deviation of model accuracy across sequential reports. This intra-patient variability, defined as the longitudinal error rate, provided a quantitative measure of model reliability and temporal stability—critical for assessing performance in time-dependent clinical scenarios.

Prior to all analyses, assumptions of normality and homogeneity of variance were verified. Additionally, inter-rater reliability for ordinal variables was assessed using Cohen’s weighted kappa coefficient with quadratic weighting, based on independent evaluations of the dataset.

## Results

The study analyzed 556 TACE reports from 50 consecutive HCC patients treated between 2012 and 2024. To ensure the reliability of the ordinal scoring, the dataset was independently evaluated by a second board-certified radiologist. Inter-rater reliability for individual variables ranged from κ = 0.68 to 0.98, see Table 1. Binary variables showed an average agreement of 92.5%, with the highest agreement observed for objective metrics such as “diagnosis accurate” (95.1%), “BCLC stage correct” (94.8%), and “Child–Pugh score correct” (95.7%). For ordinal variables, exact agreement averaged 88.5%, and 94.6% of ratings fell within ± 1. The lowest exact agreement (64.0%, with 89.6% within ± 1) occurred in the assessment of vascular invasion, reflecting the complexity and interpretative difficulty of this clinical variable.

**Table 1:** Inter-rater reliability for clinical variable extraction from TACE reports

Clinical variable	Variable type	Agreement n/N (%)	95% CI	Cohen's κ (95% CI)	*p*-value
_*Binary variables*_					
Exam dates chronological	Binary	551/556 (99.10)	97.90–99.70	0.98 (0.96–1.00)	< 0.001
TACE complications reported	Binary	550/556 (98.90)	97.60–99.60	0.97 (0.95–0.99)	< 0.001
TACE procedure dates accurate	Binary	546/556 (98.20)	96.70–99.10	0.96 (0.93–0.99)	< 0.001
Target lesions identified	Binary	545/556 (98.00)	96.50–99.00	0.96 (0.93–0.99)	< 0.001
TACE type consistency	Binary	544/556 (97.80)	96.30–98.90	0.95 (0.92–0.98)	< 0.001
mRECIST assessment correct	Binary	540/556 (97.10)	95.40–98.30	0.94 (0.91–0.97)	< 0.001
Initial diagnosis date accurate	Binary	534/556 (96.00)	94.10–97.50	0.91 (0.87–0.95)	< 0.001
Child–Pugh score correct	Binary	532/556 (95.70)	93.60–97.20	0.91 (0.87–0.95)	< 0.001
Diagnosis accurate	Binary	529/556 (95.10)	93.00–96.80	0.90 (0.86–0.94)	< 0.001
BCLC stage correct	Binary	527/556 (94.80)	92.60–96.50	0.89 (0.85–0.93)	< 0.001
Follow-up recommendations	Binary	524/556 (94.20)	92.10–95.90	0.88 (0.84–0.92)	< 0.001
TACE suitability assessment	Binary	520/556 (93.50)	91.30–95.30	0.87 (0.82–0.92)	< 0.001
Alternative treatments appropriate	Binary	512/556 (92.10)	89.60–94.10	0.84 (0.79–0.89)	< 0.001
Lipiodol retention reported†	Binary	423/467 (90.60)	87.60–93.00	0.81 (0.75–0.87)	< 0.001
AFP trend accurately reported	Binary	489/556 (87.90)	85.00–90.50	0.76 (0.69–0.83)	< 0.001
Time to progression calculated	Binary	467/556 (83.90)	80.60–86.90	0.68 (0.61–0.75)	< 0.001
Ordinal variables		Exact agreement (%)	Within ± 1 (%)	Weighted κ‡ (95% CI)	
Lymph node status	1–5 scale	534/556 (96.00)	99.30	0.94 (0.91–0.97)	< 0.001
Distant metastases identified	1–5 scale	517/556 (93.00)	98.60	0.91 (0.87–0.95)	< 0.001
Key changes summary	1–5 scale	500/556 (89.90)	97.80	0.88 (0.84–0.92)	< 0.001
Error severity assessment	1–5 scale	489/556 (87.90)	97.10	0.86 (0.82–0.90)	< 0.001
Accuracy across time points	1–5 scale	478/556 (85.90)	96.40	0.85 (0.81–0.89)	< 0.001
Liver function trends	1–5 scale	478/556 (85.90)	96.00	0.84 (0.80–0.88)	< 0.001
Liver segments identified	1–5 scale	434/556 (78.10)	91.70	0.82 (0.78–0.86)	< 0.001
Vascular involvement	1–5 scale	356/556 (64.00)	89.60	0.71 (0.66–0.76)	< 0.001
*Summary statistics*					
Overall (24 variables)	Combined	91.50	–	0.81 (0.78–0.84)	< 0.001
Binary variables (n = 16)	Binary	92.50	–	0.82 (0.79–0.85)§	< 0.001
Ordinal variables (n = 8)	1–5 scale	88.50	94.60	0.79 (0.75–0.83)‡	< 0.001

CI = confidence interval; TACE = transarterial chemoembolization; BCLC = Barcelona Clinic Liver Cancer; AF*P = *alpha-fetoprotein; mRECIST = modified Response Evaluation Criteria in Solid Tumors

^†^ Assessed in 467 reports where lipiodol was administered

^‡^ Weighted kappa with quadratic weights

^§^ Pooled kappa for binary variables

**Notes:** Two board-certified interventional radiologists independently evaluated all 556 reports across 24 clinical variables (13,344 total ratings). Disagreements were resolved through consensus (84.30%) or third reviewer consultation (15.70%). McNemar's test showed no systematic bias between raters (χ^2^ = 2.34, *p = *0.126). Overall disagreement rate = 8.5% (2,272/26,688 ratings). Each rater evaluated 24 variables × 556 reports = 13,344 ratings. Total ratings from both raters before consensus = 26,688

**Fig. 1:**
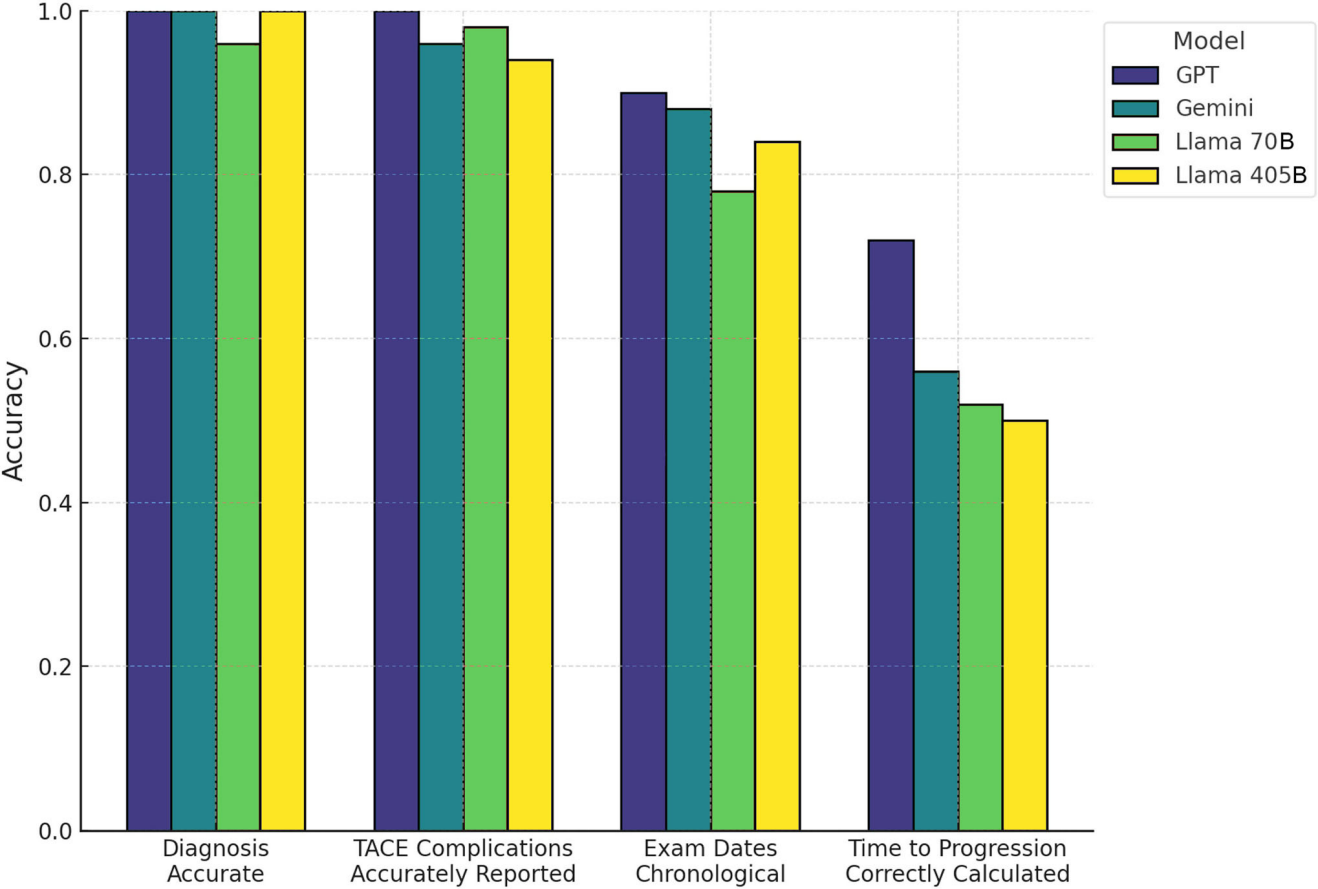
Bar plot showing mean accuracy of four large language models (GPT-4o, Gemini 1.5 Pro, Llama 3.1 70B, and Llama 3.1 405B) across representative binary tasks. Diagnosis accuracy and complication reporting were uniformly near ceiling (> 0.95) across all models. Temporal consistency in exam date ordering declined slightly, with GPT-4o and Gemini maintaining accuracies around 0.88–0.90, compared to 0.78 for Llama 3.1 70B and 0.85 for Llama 3.1 405B. The largest performance gap occurred in the time-to-progression calculation, where GPT-4o reached 0.72, Gemini 0.56, and both Llama models approximately 0.50, reflecting the higher complexity of computation-dependent assessments

**Fig. 2:**
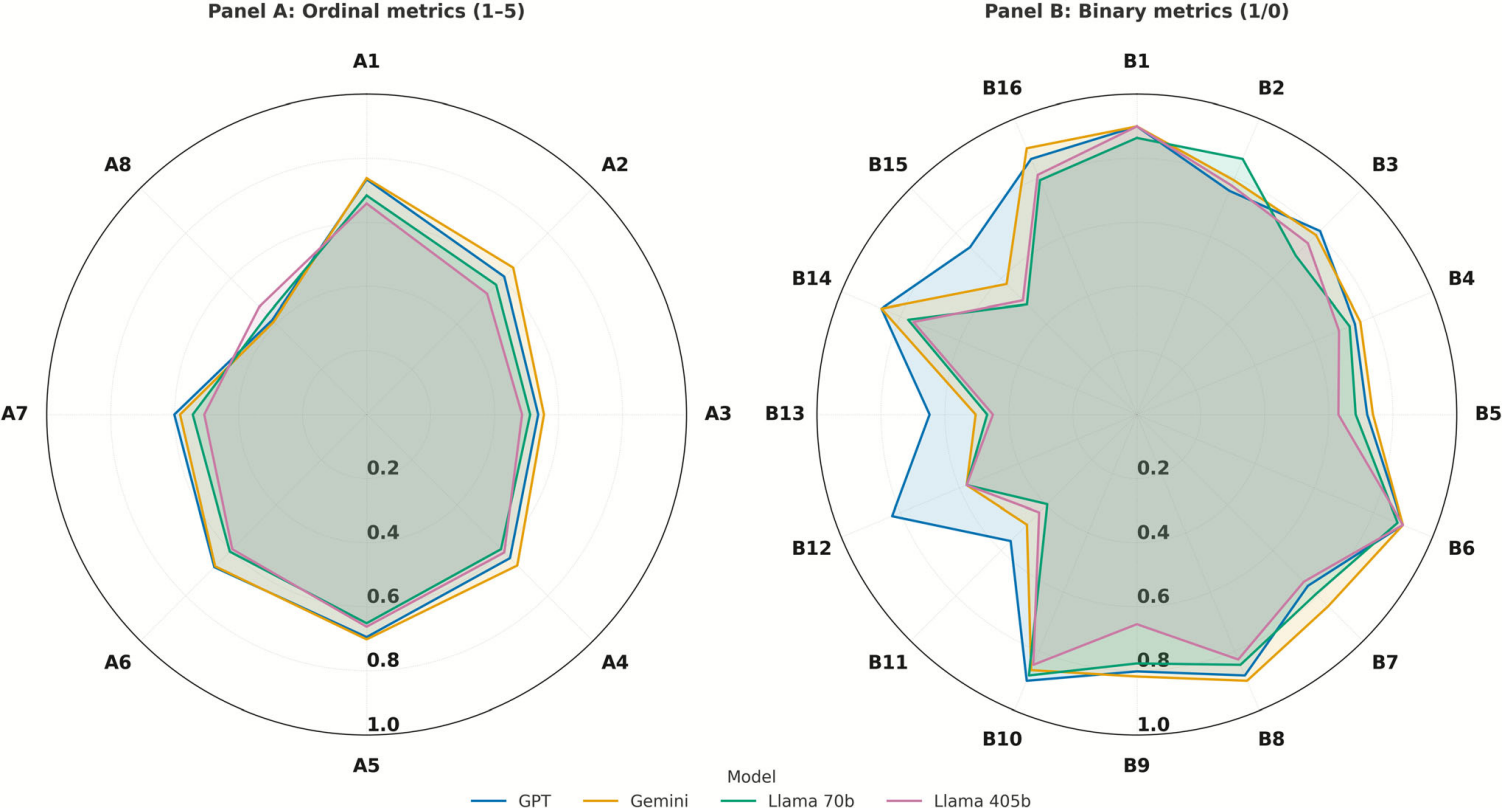
Comparative performance of four large language models in extracting structured information from TACE reports. Radial axes show normalized mean scores from 0.0 at the center to 1.0 at the periphery, with grid lines every 0.2. Polygons are scaled to ninety percent of full radius so that axis codes remain outside the plotting field. Ordinal variables (1–5 scale): A1 Liver Segments Accurately Identified, A2 Vascular Involvement Correctly Reported, A3 Lymph Node Status Accurately Described, A4 Distant Metastases Identified, A5 Accuracy of Findings Across Time Points, A6 Key Changes Summary Correct, A7 Liver Function Trends Accurately Reported, A8 Error Severity. Binary variables (1/0 scale): B1 Diagnosis Accurate, B2 Initial Diagnosis Date Accurate, B3 Exam Dates Chronological, B4 BCLC Stage Correct, B5 Child–Pugh Score Correct, B6 TACE Procedure Dates Accurate, B7 TACE Type Consistency, B8 Target Lesions Correctly Identified, B9 Lipiodol Retention Accurately Reported, B10 TACE Complications Accurately Reported, B11 mRECIST Assessment Correct, B12 AFP Trend Accurately Reported, B13 Time to Progression Correctly Calculated, B14 TACE Suitability Assessment Appropriate, B15 Alternative Treatments Appropriately Suggested, B16 follow-up Imaging Recommendations Appropriate. Ordinal scores (1–5) were normalized to 0–1 for visualization

**Fig. 3:**
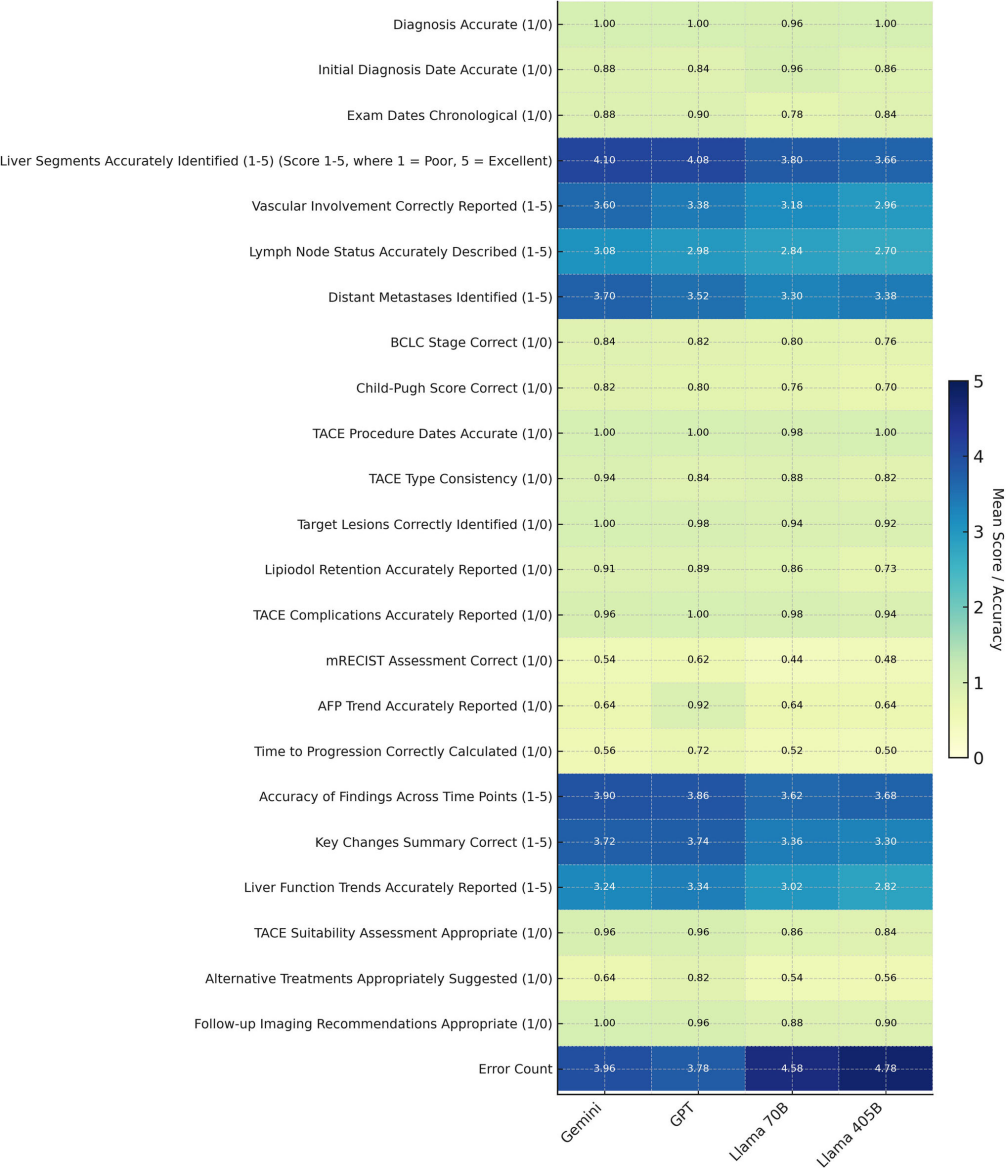
Heatmap of model performance across binary and ordinal metrics in TACE reporting. Heatmap comparing the mean performance of GPT, Gemini, Llama 3.1 70B, and Llama 3.1 405B on transarterial chemoembolization reporting tasks. Each cell shows either the mean accuracy for binary metrics scored as 1 or 0 or the mean score for ordinal metrics rated from 1 to 5. Color intensity increases from pale blue at low performance through deep blue at high performance. Binary metrics cover items such as diagnosis accuracy, initial diagnosis date accuracy, chronological exam ordering, TACE procedure date accuracy, target lesion identification, and follow-up imaging recommendation accuracy. Ordinal metrics cover liver segment identification, vascular involvement reporting, distant metastasis detection, summary of key changes, accuracy of findings over time, and liver function trend reporting. GPT and Gemini attain higher scores across most metrics. Llama 3.1 70B and Llama3.1 405B perform less well overall, with their lowest scores in liver segment identification and liver function trend reporting

The four evaluated LLMs, two proprietary models (GPT-4o and Gemini 1.5 Pro) and two open-source models (Llama 3.1 70B, Llama 3.1 405B), displayed variable accuracy and reliability in extracting key clinical data from TACE reports. Across binary and ordinal evaluations, GPT-4o and Gemini consistently outperformed both Llama models, demonstrating superior accuracy, consistency, and efficiency (Figs. 1, 2, and 3).

## Overall Model Performance

### Ordinal Variables

For ordinal variables, which were evaluated on a 1–5 scale, similar trends were observed (Fig. 2). GPT scored the highest overall (4.15, SD = 0.34), followed by Gemini (4.10, SD = 0.36). In comparison, the open-source models scored lower: Llama 3.1 70B achieved a mean score of 3.77 (SD = 0.43), and Llama 3.1 405B performed the worst with a mean score of 3.65 (SD = 0.45). ANOVA analysis showed significant differences among the models (F-statistic = 10.89, *p < *0.001). GPT and Gemini were significantly better than both Llama models (*p < *0.05 for all pairwise comparisons).

### Binary Variables

Mean performance scores for binary variables revealed significant differences among the models (Fig. 1). GPT achieved the highest mean accuracy (0.870, SD = 0.214), followed closely by Gemini (0.847, SD = 0.163). These proprietary models demonstrated strong capabilities in accurately extracting binary clinical data. In contrast, the open-source models underperformed: Llama 3.1 70B achieved a mean score of 0.798 (SD = 0.218), while Llama 3.1 405B had the lowest performance with a mean score of 0.773 (SD = 0.240). Statistical analysis using a one-way ANOVA confirmed significant differences among the models (F-statistic = 11.98, *p < *0.001). Post hoc Tukey’s HSD testing revealed that GPT and Gemini significantly outperformed Llama 3.1 405B (*p < *0.05). The GPT vs Gemini comparison also reached significance (*p = *0.048; adjusted contrast = 0.023, 95% CI 0.001–0.045; effect size r = 0.22), favoring GPT.

## Task-specific Performance

### Liver Segment Involvement

GPT and Gemini excelled in identifying liver segment involvement, achieving mean scores of 4.08 (GPT: SD = 0.34) and 4.10 (Gemini: SD = 0.36) respectively. In contrast, the open-source models underperformed, with Llama 3.1 70B scoring 3.80 (SD = 0.42) and Llama 3.1 405B scoring 3.66 (SD = 0.44), indicating frequent omissions or misidentifications of liver segments critical for TACE planning.

### Vascular Involvement

Detection of vascular invasion, particularly portal vein involvement, showed similar trends. Gemini achieved the highest accuracy (3.60, SD = 0.36), followed by GPT (3.38, SD = 0.37). The Llama models showed poorer performance, with Llama 3.1 70B scoring 3.18 (SD = 0.40) and Llama 3.1 405B scoring 2.96 (SD = 0.42).

### mRECIST Assessment

For mRECIST assessment, which evaluates tumor response to TACE, GPT achieved the highest score (4.28, SD = 0.31), followed by Gemini (4.22, SD = 0.33). The Llama models showed lower performance, with Llama 3.1 70B scoring 3.78 (SD = 0.41) and Llama 3.1 405B scoring 3.70 (SD = 0.44).

### AFP Trend Reporting

GPT-4o (0.92) significantly outperformed the other models, which all achieved 0.64. These differences highlight the superior performance of GPT-4o in handling the nuances of AFP trend extraction, which is critical for monitoring disease progression and informing treatment decisions in HCC management.

## Longitudinal Error Analysis

**Fig. 4:**
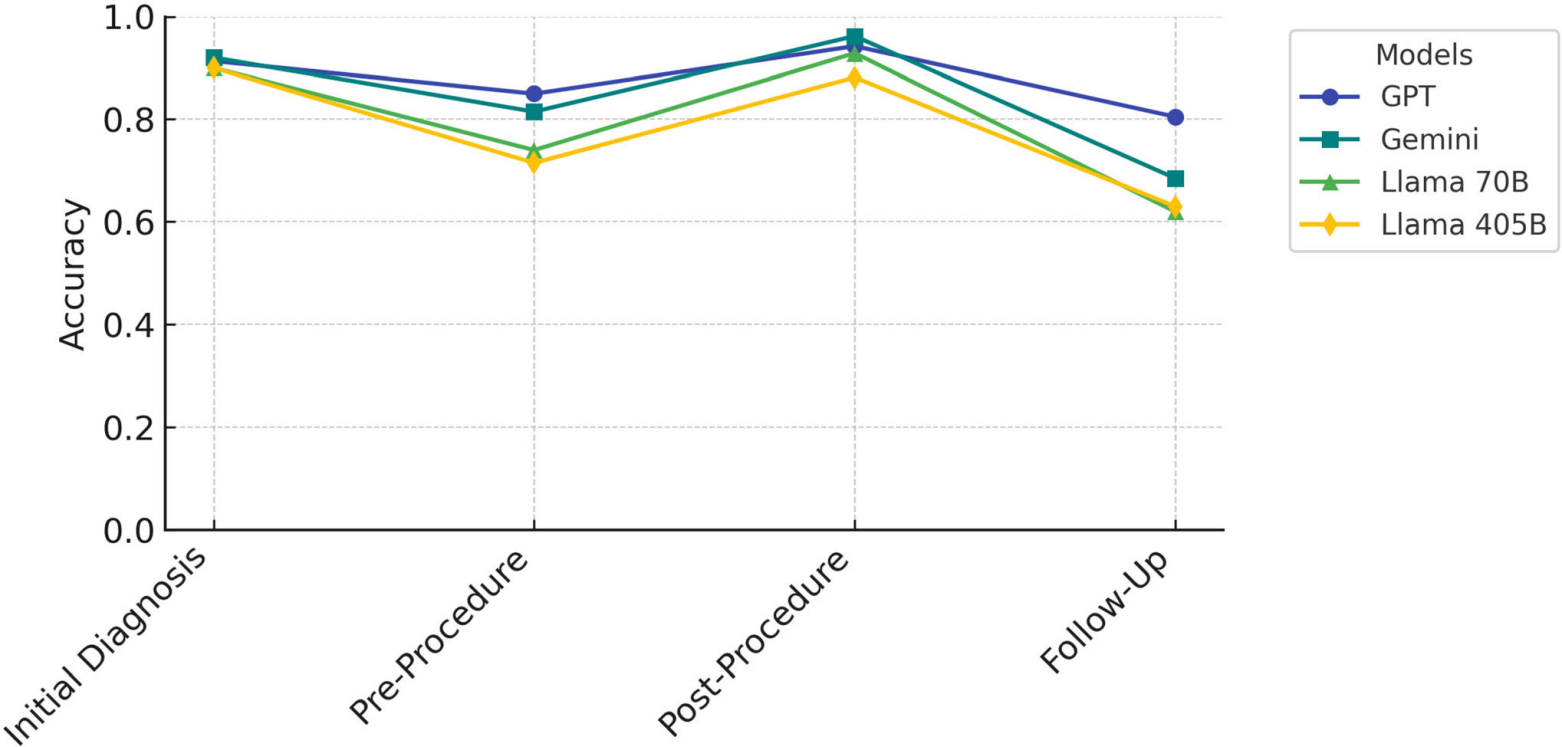
Model performance across key categories in TACE reporting. Comparative accuracy of four large language models, GPT (dark blue), Gemini (teal), Llama 3.1 70b (lime), and Llama 3.1 405b (yellow), across four TACE reporting phases: Initial Diagnosis (diagnosis correctness and date accuracy), Pre-Procedure Assessment (Child–Pugh score, BCLC stage, suitability), Post-Procedure Documentation (complications, mRECIST response, AFP trend, time to progression), and Follow-Up Evaluation (imaging recommendations, alternative treatments). Each point represents the mean of all binary metrics within that phase. GPT and Gemini sustain high accuracy (> 0.90) through Initial Diagnosis and Pre-Procedure, whereas both Llama models drop sharply after Pre-Procedure. All models recover partially at Post-Procedure but fall again at Follow-Up, with Llama 3.1 405b showing the lowest overall performance

**Fig. 5:**
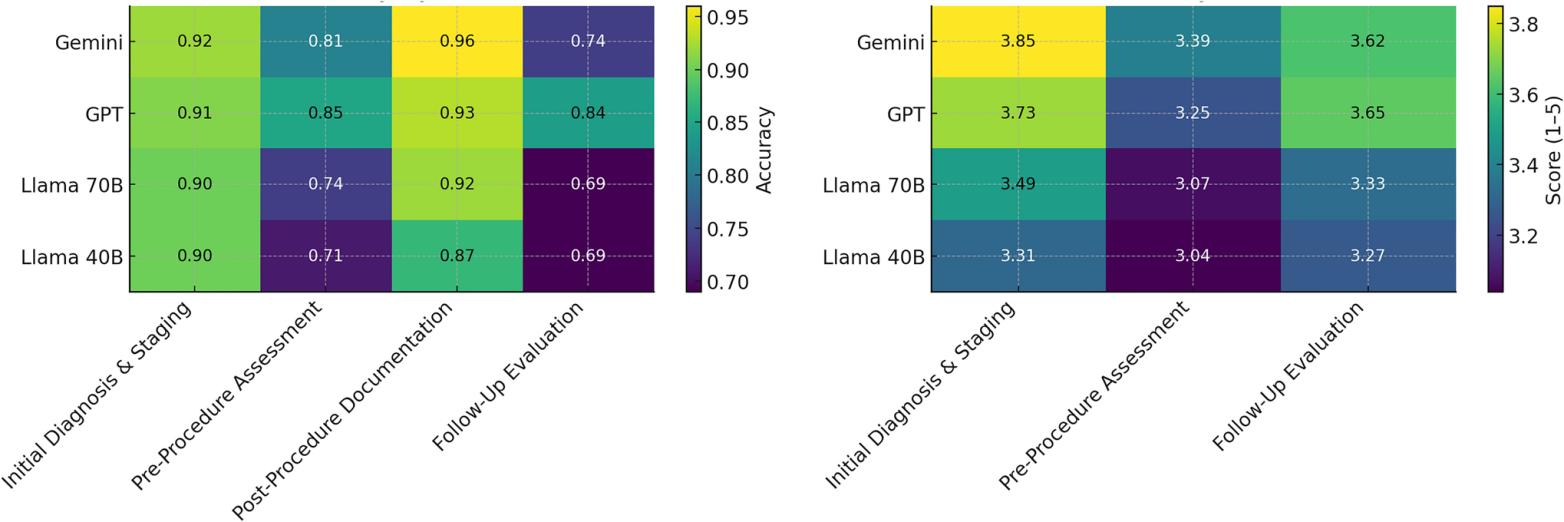
Comparative heatmaps of model performance across reporting categories. This figure presents two side‐by‐side heatmaps of model performance across TACE reporting phases. The left panel maps mean accuracy (0–1) of binary metrics: Initial Diagnosis & Staging, Pre-Procedure Assessment, Post-Procedure Documentation, Follow-Up Evaluation—for GPT, Gemini, Llama 3.1 70b and Llama 3.1 405b. Both GPT (0.91) and Gemini (0.92) exceed 0.90 in Initial Diagnosis & Staging, while Llama models decline to 0.69 in Follow-Up Evaluation. The right panel displays mean ordinal scores (1–5 normalized to raw averages) for three phases—Initial Diagnosis & Staging, Pre-Procedure Assessment, and Follow-Up Evaluation, with Gemini leading at 3.85, GPT at 3.73, and Llama 3.1 70b/405b trailing most notably in Pre-Procedure (3.07/3.04) and Follow-Up (3.33/3.27), indicating challenges with complex assessment and follow-up tasks

A longitudinal error analysis revealed notable variability in model performance across sequential reports. Among binary variables, GPT demonstrated the lowest mean error rate (0.01, SD = 0.10) followed by Gemini (0.09, SD = 0.12). In contrast, the open-source models exhibited higher error rates, with Llama 3.1 70B showing a mean error rate of 0.18 (SD = 0.12) and Llama 3.1 405B exhibiting the highest error rate of 0.21 (SD = 0.14). For ordinal variables, GPT and Gemini also showed the least error variability, with SDs of 0.11 and 0.12, respectively, compared to Llama 3.1 70B (SD = 0.14) and Llama 3.1 405B (SD = 0.15) (Figs. 4 and 5).

## Discussion

This study highlights the potential of LLMs, particularly proprietary systems such as Gemini and GPT, in advancing interventional oncology. These models demonstrated robust performance in extracting critical data from TACE reports, excelling in areas such as liver segment involvement, mRECIST tumor response evaluation, and AFP biomarker trend analysis—key variables for managing HCC (27–30). Proprietary models consistently outperformed open-source alternatives, such as Llama 3.1 70B and Llama 3.1 405B, across all evaluated metrics. The ability of proprietary models to accurately interpret these key clinical factors supports the integration of LLMs into clinical workflows, enhancing decision-making and optimizing treatment strategies in interventional oncology. However, limitations in certain tasks, such as vascular invasion detection, underscore the boundaries of current AI capabilities in clinical decision-making (29, 30).

Proprietary models demonstrated superior accuracy in tasks such as liver segment identification and longitudinal AFP trend analysis. These higher scores of the proprietary models indicate fewer omissions or misidentifications of liver segments, which is critical for accurate TACE planning, when compared to the lower-performing open-source models. Additionally, the proprietary models exhibited lower longitudinal error rates, highlighting their exceptional consistency and stability in processing sequential clinical data. This underscores their reliability for tasks that require longitudinal accuracy and precision, reinforcing their potential to support routine clinical workflows with enhanced efficiency and decision-making accuracy (27). In addition to improving accuracy, these models can streamline routine workflows by automating repetitive tasks, enabling clinicians to focus on more complex decision-making processes. Despite their strengths, proprietary models showed slight limitations with vascular invasion detection. Vascular invasion is essential in determining treatment eligibility and involves highly variable and descriptive documentation. Even with advanced architectures, these models were unable to overcome the lack of standardization in clinical reports, highlighting the need for more consistent and structured reporting practices to enhance model accuracy and reliability (14). Standardized reporting templates could ensure uniformity in the documentation of critical variables, such as vascular invasion, making it easier for AI models to interpret and extract relevant information. Without such standardization, even the most advanced models remain limited by inconsistencies in language, terminology, and data structure, which can compromise their reliability and hinder clinical decision-making. Open-source models, such as Llama 3.1 70B (3.77/5) and Llama 3.1 405B (3.65/5), demonstrated weaker performance, particularly in complex and nuanced tasks such as vascular invasion detection. Interestingly, larger open-source models (Llama 3.1 405B) did not consistently outperform smaller ones (Llama 3.1 70B), challenging the assumption that higher parameter count guarantees better performance. This underscores the importance of domain-specific alignment, optimization, and targeted training rather than sheer model size. Furthermore, although statistically significant performance differences were observed, their clinical relevance must be interpreted cautiously. Small numerical improvements (e.g., a 0.3-point difference on a 5-point ordinal scale) may not translate into meaningful changes in clinical practice.

To enhance open-source model performance, collaborative efforts are needed to develop comprehensive, publicly accessible repositories of anonymized clinical data. Such initiatives would help bridge current performance gaps, making open-source solutions more viable in diverse clinical environments (16, 20, 27).

The most significant limitation across all evaluated models is their reliance exclusively on textual and tabular data. In clinical practice, decision-making, particularly regarding tasks such as vascular invasion detection, heavily depends on integrated analysis of textual, tabular, and imaging data. Therefore, future research should focus on developing multimodal AI systems capable of synthesizing diverse data types, closely mirroring clinicians’ holistic interpretative strategies.

Moreover, the performance of AI models is substantially limited by variability in clinical documentation. Introducing standardized reporting protocols could significantly reduce variability, enhance AI training data quality, and improve overall clinical communication and decision-making processes (14). Inter-rater reliability assessment confirmed excellent agreement (κ = 0.81), reinforcing the validity of the ground truth annotations used to evaluate model performance.

Expanding training datasets to include diverse, real-world clinical cases is another crucial step toward improving AI model performance. Incorporating heterogeneous datasets encompassing both straightforward and complex scenarios would enhance the robustness and generalizability of AI models. This approach ensures that AI systems are equipped to handle the full spectrum of clinical challenges, enabling them to perform effectively across various settings and patient populations. By training models on more representative data, we can achieve greater reliability and adaptability in real-world applications.

The integration of multimodal data, the standardization of clinical documentation, and the expansion of training datasets represent critical pathways for advancing AI in interventional oncology and potentially for tumor board workflows. Collaborative efforts between healthcare institutions, AI developers, and academic researchers are essential for addressing the technical and ethical challenges identified in this study (29–35). Furthermore, ethical considerations, such as ensuring patient data confidentiality, and practical challenges, including the seamless integration of AI models into clinical workflows, must be carefully addressed to ensure their successful and responsible implementation (30, 33–35). Open-source models, while currently less competitive, could be significantly improved through targeted training and broader access to high-quality clinical data. Although the results demonstrate technical feasibility, the clinical applicability remains speculative in the absence of outcome validation or integration into real-world decision-making workflows. While proprietary models such as Gemini and GPT demonstrate strong capabilities for automating the extraction of critical interventional radiology–specific variables (e.g., liver segment involvement, mRECIST response, AFP trends), their integration into clinical decision-making workflows, particularly multidisciplinary tumor boards, requires careful consideration. Such boards typically incorporate extensive clinical, pathological, and imaging data beyond IR reports alone. Hence, the effectiveness of these LLM-generated summaries depends heavily on the comprehensiveness and consistency of clinical documentation.

## Limitations

This study has several notable limitations. Firstly, the exclusive reliance on textual and tabular data without incorporation of imaging restricts comprehensive clinical assessments, likely limiting performance in complex interpretive tasks such as vascular invasion detection.

Secondly, the exclusion of reports with incomplete or ambiguous documentation ensured consistent ground truth but may lead to an overestimation of real-world model performance, where incomplete documentation frequently occurs. Future research should assess AI performance under realistic conditions involving ambiguous or incomplete data.

Thirdly, the quality and comprehensiveness of clinical documentation directly impact AI performance. LLMs cannot compensate for undocumented clinical details that are critical for multidisciplinary tumor board decisions.

Fourthly, data from a single referral center potentially limit the generalizability of results to other institutions with varying documentation practices and patient demographics.

Finally, reliance on proprietary training data restricts model transparency and wider accessibility, underscoring the need for improved open-source models and multi-institutional data validation. Future studies should incorporate imaging modalities, expand multi-institutional datasets, and explore parameter tuning effects to refine performance comparisons between proprietary and open-source models.

## Conclusion

Proprietary large language models such as GPT-4o and Gemini demonstrate substantial promise for automating clinical information extraction from TACE reports, enhancing workflow efficiency, and supporting decision-making in interventional oncology. Nevertheless, current AI models, including proprietary ones, still face challenges with complex interpretive tasks such as vascular invasion detection, underscoring the continued necessity for human oversight and clinical validation.

Open-source models, despite current performance gaps, offer significant advantages in transparency, adaptability, and broader accessibility. Their ongoing refinement through targeted training, multimodal data integration, and standardized clinical documentation practices remains critical. Ultimately, successful clinical integration of AI should focus on collaborative development, transparent validation, and adherence to ethical standards, ensuring that AI complements rather than replaces essential human judgment in patient care. Further prospective, outcome-oriented studies are required to fully validate the clinical utility of these promising technologies.

## Supplementary Information

Below is the link to the electronic supplementary material.

Supplementary file1 (DOCX 24 KB)

## Data Availability

The datasets used and/or analyzed during the current study are available from the corresponding author on reasonable request.
