# Supplementary material for: Comparative Evaluation of Proprietary and Open-Source Large Language Models for Systematic Multi-source Information Extraction in Interventional Oncology
Source: Cardiovasc Intervent Radiol. 2025 Dec 7;49(5):992–1004. doi: 10.1007/s00270-025-04287-1 (PMC13156197; doi:10.1007/s00270-025-04287-1)
Supplement: Supplementary file 1 — Supplementary file1 (DOCX 24 KB) [file 270_2025_4287_MOESM1_ESM.docx]

**Supplement 1: TACE REPORT PROMPT & TEMPLATE**

You are provided with clinical documentation from a patient who underwent TACE for hepatocellular carcinoma. Please extract all relevant clinical information and organize it according to the following template. For each field marked with [Extract] or containing bracketed options like [Y/N], identify and populate the corresponding information from the provided reports. If information is not available for a field, indicate 'Not documented'. Maintain chronological order for all temporal data:

**I. PATIENT DEMOGRAPHICS & BASELINE**

- Patient ID: [Extract]
- Age/Sex: [Extract] / [M/F]
- Initial HCC Diagnosis Date: [Extract]
- Etiology: [HBV/HCV/NASH/Alcohol/Other]
- Baseline ECOG: [0-4]
- Baseline Child-Pugh: [Extract]
- Baseline BCLC Stage: [Extract]

**II. TREATMENT TIMELINE**

| Date | Imaging | BCLC | Child-Pugh | TACE (Y/N) | AFP | Comments |
| --- | --- | --- | --- | --- | --- | --- |
| [Extract] | [CT/MRI] | [0-D] | [A5-C15] | [Y/N] | [Value] | [Extract] |

**III. CURRENT HEPATIC STATUS**

Date of Assessment: [Extract]

- Cirrhosis: [Present/Absent]
- Morphology: [Micro/Macronodular]
- Liver Volume: [Normal/Reduced/Enlarged] - [cc value]
- Ascites: [None/Trace/Small/Moderate/Large]
- Varices: [None/Small/Large] - Location: [Extract]
- Portal Hypertension: [None/Mild/Moderate/Severe]
- Spleen Size: [cm]

**IV. TUMOR ASSESSMENT**

Target Lesions

| ID | Segment | Size (mm) | LI-RADS | Enhancement | Washout | Capsule |
| --- | --- | --- | --- | --- | --- | --- |
| L[#] | [I-VIII] | [Extract] | [3-5/M] | [Y/N] | [Y/N] | [Y/N] |

Non-Target Lesions

- Total Number: [#]
- Segments Involved: [List segments]
- Largest Non-Target: [Size] mm in segment [Location]

Overall Tumor Burden: [<25% / 25-50% / >50%] of liver

**V. VASCULAR ASSESSMENT**

| Vessel | Status |
| --- | --- |
| Main Portal Vein | [Patent/Partial thrombosis/Complete occlusion] |
| Right Portal Vein | [Patent/Invaded/Thrombosed] |
| Left Portal Vein | [Patent/Invaded/Thrombosed] |
| Hepatic Veins | [Patent/Compressed/Invaded] |
| Hepatic Artery | [Normal/Variant anatomy/Stenosis] |
| Collaterals | [Absent/Present]: [Describe if present] |

**VI. EXTRAHEPATIC DISEASE**

- Lymph Nodes: [None/Regional/Distant] - Size/Location: [Extract]
- Lungs: [No metastases/Metastases present] - Details: [Extract]
- Bones: [No metastases/Metastases present] - Details: [Extract]
- Other: [Extract]

**VII. TACE PROCEDURE DETAILS**

Procedure Date: [Extract] TACE Number: #[Extract]

- Type: [cTACE/DEB-TACE]
- Vascular Access: [Extract]
- Target Vessels: [Extract]
- Chemotherapy: [Drug] [Dose] mg
- Embolic Agent: [Extract]
- Technical Success: [Complete/Partial/Failed]
- Immediate Complications: [None/List]

**VIII. TREATMENT RESPONSE**

Per Lesion (mRECIST)

| Lesion | Pre-TACE Size | Post-TACE Size | Enhancement | Response |
| --- | --- | --- | --- | --- |
| L[#] | [Extract] | [Extract] | [Y/N] | [CR/PR/SD/PD] |

Overall Response

- Best Response Achieved: [CR/PR/SD/PD]
- Time to Progression: [#] months
- Pattern of Progression: [None/Local/New intrahepatic/Extrahepatic]

**IX. LABORATORY VALUES**

| Test | Baseline | Current | Trend |
| --- | --- | --- | --- |
| AFP (ng/mL) | [Value] | [Value] | [↑/↓/→] |
| Bilirubin (mg/dL) | [Value] | [Value] | [↑/↓/→] |
| Albumin (g/dL) | [Value] | [Value] | [↑/↓/→] |
| INR | [Value] | [Value] | [↑/↓/→] |
| Platelets (K/μL) | [Value] | [Value] | [↑/↓/→] |
| Creatinine (mg/dL) | [Value] | [Value] | [↑/↓/→] |

**X. CURRENT CLINICAL STATUS**

- BCLC Stage: [0/A/B/C/D]
- Child-Pugh Score: [Score] (Class [A/B/C])
- ECOG Performance Status: [0-4]
- MELD Score: [#]
- ALBI Grade: [1/2/3]

**XI. RECOMMENDATIONS**

Immediate Plan:

[Select appropriate option]

- Continue TACE - Next session in [#] weeks
- Switch to Systemic Therapy: [Specify drugs]
- Consider Y90 Radioembolization
- Transplant Evaluation
- Best Supportive Care
- Clinical Trial Enrollment

Follow-up:

- Next Imaging: [MRI/CT] in [#] weeks
- Lab Work: [Frequency and tests]
- Clinic Visit: [Timeframe]

**XII. SUMMARY**

[Extract narrative summary including: disease trajectory, response to TACE, current challenges, prognosis, and rationale for recommendations]
